# Infants and Air

**Published:** 1876-05

**Authors:** 


### INFANTS AND AIR.

PARLIAMENTARY returns show that of twenty-eight hundred infants annually sent to various hospitals to be taken care of, twenty-four out of twenty-five died before they were a year old! A law was immediately passed that they should be sent to the country thereafter, when it was found that only nine out of twenty-five died the first year; that is, instead of twenty-six hundred and ninety dying, there were only four hundred and fifty, a difference of twenty-two hundred and forty.

